# AmyZ1: a novel α-amylase from marine bacterium *Pontibacillus* sp. ZY with high activity toward raw starches

**DOI:** 10.1186/s13068-019-1432-9

**Published:** 2019-04-23

**Authors:** Wei Fang, Saisai Xue, Pengjun Deng, Xuecheng Zhang, Xiaotang Wang, Yazhong Xiao, Zemin Fang

**Affiliations:** 1 School of Life Sciences, Anhui University, Hefei, 230601 Anhui China; 2 Anhui Key Laboratory of Modern Biomanufacturing, Hefei, 230601 Anhui China; 3 Anhui Provincial Engineering Technology Research Center of Microorganisms and Biocatalysis, Hefei, 230601 Anhui China; 4 Department of Chemistry & Biochemistry, Florida International University, Miami, FL 33199 USA

**Keywords:** α-Amylase, Specific activity, Raw starch hydrolysis, *Pontibacillus* sp.

## Abstract

**Background:**

Starch is an inexpensive and renewable raw material for numerous industrial applications. However, most starch-based products are not cost-efficient due to high-energy input needed in traditional enzymatic starch conversion processes. Therefore, α-amylase with high efficiency to directly hydrolyze high concentration raw starches at a relatively lower temperature will have a profound impact on the efficient application of starch.

**Results:**

A novel raw starch digesting α-amylase (named AmyZ1) was screened and cloned from a deep-sea bacterium *Pontibacillus* sp. ZY. Phylogenetic analysis showed that AmyZ1 was a member of subfamily 5 of glycoside hydrolase family 13. When expressed in *Escherichia coli*, the recombinant AmyZ1 showed high activity at pH 6.0–7.5 and 25–50 °C. Its optimal pH and temperature were 7.0 and 35 °C, respectively. Similar to most α-amylases, AmyZ1 activity was enhanced (2.4-fold) by 1.0 mM Ca^2+^. Its half-life time at 35 °C was also extended from about 10 min to 100 min. In comparison, AmyZ1 showed a broad substrate specificity toward raw starches, including those derived from rice, corn, and wheat. The specific activity of AmyZ1 towards raw rice starch was 12,621 ± 196 U/mg, much higher than other reported raw starch hydrolases. When used in raw starch hydrolyzing process, AmyZ1 hydrolyzed 52%, 47% and 38% of 30% (w/v) rice, corn, and wheat starch after 4 h incubation. It can also hydrolyze marine raw starch derived from *Chlorella pyrenoidosa*, resulting in 50.9 mg/g DW (dry weight of the biomass) of reducing sugars after 4 h incubation at 35 °C. Furthermore, when hydrolyzing raw corn starch using the combination of AmyZ1 and commercial glucoamylase, the hydrolysis rate reached 75% after 4.5 h reaction, notably higher than that obtained in existing starch-processing industries.

**Conclusions:**

As a novel raw starch-digesting α-amylase with high specific activity, AmyZ1 efficiently hydrolyzed raw starches derived from both terrestrial and marine environments at near ambient temperature, suggesting its application potential in starch-based industrial processes.

**Electronic supplementary material:**

The online version of this article (10.1186/s13068-019-1432-9) contains supplementary material, which is available to authorized users.

## Background

As one of the most abundant storage of carbohydrates, starch has been extensively employed as an inexpensive and renewable raw material for food, pharmaceutical, and bio-fuel industries [1]. Due to the polycrystalline structure in native starch granules [2], enzymatic hydrolyzation at a higher temperature is required to efficiently disrupt the native starch structure in starch-based industrial processes. Briefly, raw starch is sequentially gelatinized at about 100 °C, liquefied with thermophilic α-amylases at about 95 °C, and treated with glucoamylases at 50–60 °C [3, 4]. Apparently, existing starch processing techniques are energy-intensive, and thus it is necessary to develop more efficient and energy-effective processes. To solve this problem, α-amylases capable of directly liquefying raw starch to glucose would have significant industrial implications.

α-Amylase (EC 3.2.1.1) is one of the oldest and most important industrial enzymes used for starch processing [1]. The usage of α-amylase digesting raw starch brings about 10–20% reduction in energy consumption compared to the traditional physical/chemical processes [3, 5]. However, only approximately 10% of the known α-amylases can efficiently digest raw starches directly to glucose because of their densely compacted architecture, large size, and unique surface profile [2, 3]. Furthermore, although many α-amylases with raw starch hydrolyzing activity have been identified and characterized from bacteria and fungi, few of them possess high specific activity toward raw starches [6–9]. For example, the enzymes from *Bacillus acidicola* and *Bacillus amyloliquefaciens* exhibited 793 U/mg and 45 U/mg toward raw corn starch [10, 11]. Other α-amylases such as those from *Geobacillus thermoleovorans* [12], *Rhizopus oryzae* [13], and *Thermomyces dupontii* [14] possessed specific activities of no more than 2800 U/mg. Therefore, it is essential to explore novel α-amylases with higher specific activity toward raw starches to decrease the dosage and cost on the enzyme [15].

Another factor that hampers the application of α-amylases is the low hydrolyzation efficiency towards high concentration raw starches. Typically, starch-processing industries employ 20–30% (w/v) starch slurries [16]. Systematic studies with the enzymes from *Bacillus licheniformis* [16, 17], *B. amyloliquefaciens* [12], *Bacillus subtilis* [4, 17], and *G. thermoleovorans* [12, 18] have demonstrated that improved hydrolysis toward high concentration raw starches can be achieved by increasing the incubation temperature to 60 °C or higher [19]. An alternative strategy to obtain better hydrolysis is to extend the reaction time at lower temperatures [16]. However, neither increasing temperature nor extending incubation time helps to reduce energy consumption and lower the cost of manufacturing. As a result, it is essential to explore novel α-amylases that hydrolyze high concentration raw starches with high efficiency at a lower temperature.

Due to the complexity and diversity of the marine environment, the microbes in oceans are recognized as a tremendous treasure for the discovery of novel enzymes with unique properties. Several α-amylases have been identified from the marine bacteria, including *Bacillus aquimaris* MKSC 6.2 [20], *Geobacillus* sp. 4j [17], *B. subtilis* S8–18 [4], *Halothermothrix orenii* [21], *Aeromonas salmonicida* [22], and a marine bacterial metagenome [2]. In this study, a bacterial strain with amylolytic activity was screened out from the sediment of Yongxing island and was named as *Pontibacillus* sp. ZY. A novel α-amylase coding gene, designated as *AmyZ1*, was successfully cloned from *Pontibacillus* sp. ZY and heterologously expressed in *Escherichia coli*. The recombinant enzyme AmyZ1 exhibited high specific activity and broad substrate specificity towards raw starches. Furthermore, AmyZ1 could efficiently hydrolyze high concentration raw starches at temperatures significantly lower than that used in current starch processing.

## Results and discussion

### Screening for strains producing starch hydrolyzing enzymes

After incubation at 15 °C for 3 days, approximately 3600 colonies grew on the screen plates containing soluble starch. About 200 strains showed a halo around the colonies when the plates were stained with Lugol’s iodine solution and were recognized as positive clones. One strain named ZY was chosen for further research because of its larger halo than other colonies.

Phylogenetic analysis suggested that the 16S rRNA gene of strain ZY showed 99% sequence identity to the marine bacteria *Pontibacillus halophilus* JSM 076056. Thus, this positive strain was named as *Pontibacillus* sp. ZY. *Pontibacillus* sp., implicating “*Bacillus* affiliated with the marine”, was a novel genus identified for the first time in 2005 [23–25]. By 2018, only seven species were allocated to this genus (http://www.ezbiocloud.net/). Several novel enzymes have been identified and characterized from *Pontibacillus* sp., including protease and cytochrome P450 [26]. In comparison, only one amylase was partially purified from *Pontibacillus* sp. [27]. In this context, it is meaningful to characterize the properties of α-amylase from the genus.

### Sequence analysis of AmyZ1

A gene of 1521 bp, named *AmyZ1*, was cloned from *Pontibacillus* sp. ZY. The deduced sequence encoded by *AmyZ1* contained a signal peptide comprising of 21 amino acid residues as predicted by SignalP and simple modular architecture research tool (SMART). The occurrence of a secretion signal in the deduced sequence was in agreement with the fact that AmyZ1 was secreted as an extracellular soluble protein in *Pontibacillus* sp. ZY. AmyZ1 showed the highest sequence identity of 99% (99% similarity) with the α-amylase from *P. halophilus* JSM 076056, followed by 78% identity (88% similarity) with that from *Pontibacillus chungwhensis*. Both the enzymes were deduced from the whole-genome sequencing and have not been biochemically characterized previously.

AmyZ1 contained four conserved regions that are the typical characteristics of the glycoside hydrolase family 13 (GH13) [28] (Additional file 1: Figure S1). The catalytic triad of Asp234, Glu264, and Asp331 were located in regions I, II, and III, respectively (Additional file 1: Figure S1). Based on the phylogenetic analysis, AmyZ1 was branched together with the enzymes from subfamily 5 of GH13 (GH13_5) (Additional file 2: Figure S2). In fact, AmyZ1 was recorded by CAZy as the first GH13_5 α-amylase that derived from the genus *Pontibacillus*. The GH13_5 mainly contains liquefying α-amylases from different marine bacterial sources, such as *Bacillus* sp. YX-1, *B. amyloliquefaciens*, *Bacillus cereus* and *B. licheniformis* NH1 [28, 29]. In addition, GH13_5 also contains the typical terrestrial α-amylase from *B. licheniformis* isolated from soil. The AmyZ1 structure was obtained by homology modeling using the α-amylase from *B. licheniformis* (PDB code: 1BLI, shared 71% identity and 83% similarity with AmyZ1) as the template. Results showed that AmyZ1 was comprised of three domains, including catalytic domain A, followed by domain B and C (Additional file 3: Fig. S3).

### Expression and refolding of AmyZ1

**Fig. 1:**
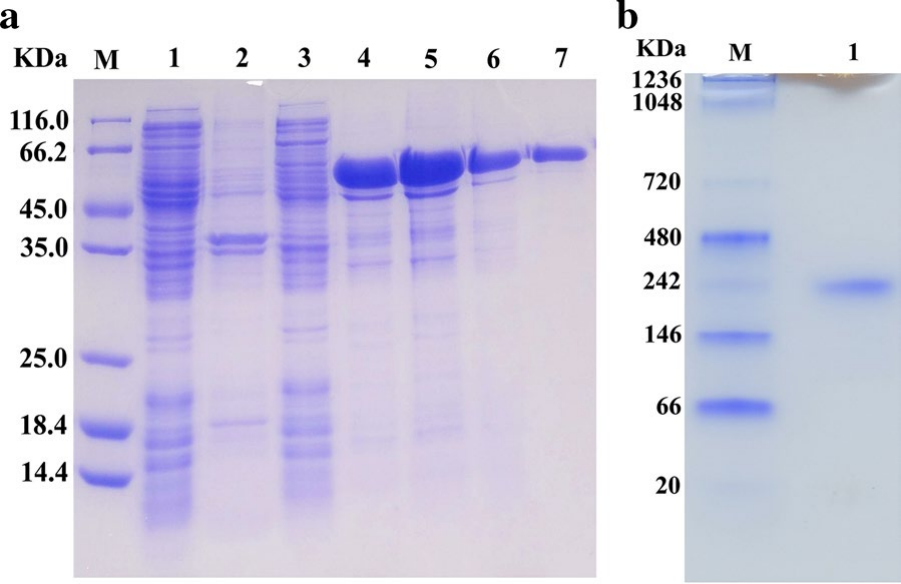
SDS-PAGE and Native-PAGE analysis of AmyZ1. **a** SDS-PAGE. M: protein molecular weight marker (Thermo Fisher Scientific, Inc.); lane 1 and 2: the sonication supernatant and precipitate of *E. coli* harboring plasmid pET22b(+)-AmyZ1 without induction; lane 3 and 4: the sonication supernatant and precipitate of *E. coli* harboring plasmid pET22b(+)-AmyZ1 induced by IPTG; lane 5: the protein denaturated by 8 M urea; lane 6: the protein after renaturation; lane 7: the target protein after dialysis. **b** Native-PAGE analysis of the purified AmyZ1. M: native protein molecular weight marker (Thermo Fisher Scientific, Inc.); lane 1: the purified native protein

The recombinant enzyme AmyZ1 was expressed as inclusion bodies even after expression condition optimization, including the initial induction *OD*_600_, isopropyl β-D-1-thiogalactopyranoside (IPTG) concentration, induction time, and incubation temperature. Fortunately, AmyZ1 can be easily refolded to its active form following the protocol described in “Methods” section. As shown in Additional file 4: Table S1, AmyZ1 was 1.3-fold purified to homology with 29.4% recovery. The purified enzyme displayed a single band on sodium dodecyl sulfate polyacrylamide gel electrophoresis (SDS-PAGE) (Fig. 1a), with an apparent molecular weight of about 55 kDa, consistent with the theoretical value calculated based on the amino acid sequence. Native-PAGE showed that AmyZ1 exhibited a molecular weight of about 240 kDa, that the active protein is a homotetramer (Fig. 1b).

### Biochemical characterization of AmyZ1

**Fig. 2:**
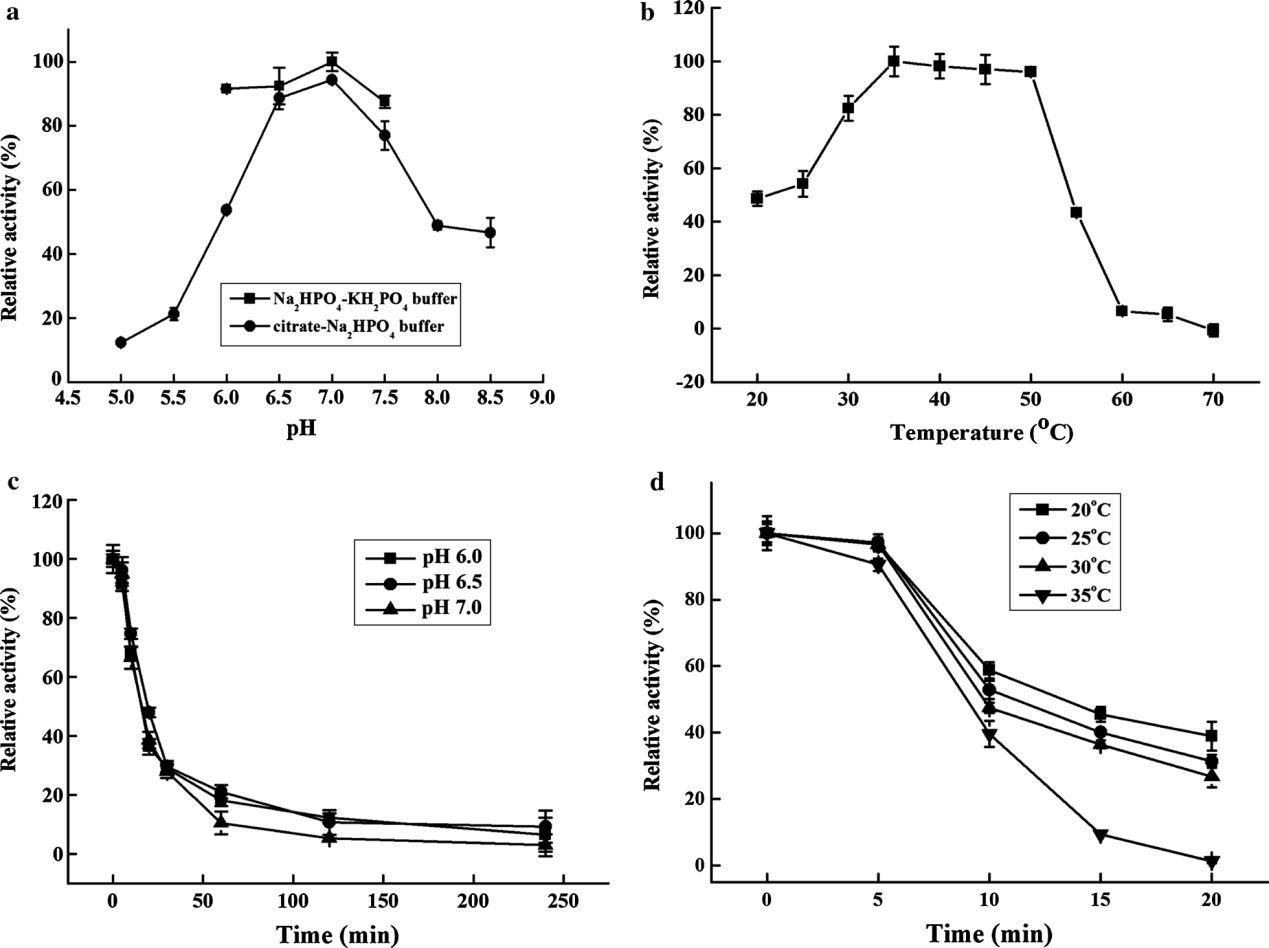
Effects of pH and temperature on AmyZ1 activity (**a**, **b**) and stability (**c**, **d**). **a** Optimum pH of AmyZ1. With raw rice starch as the substrate, the optimum pH was determined in citrate–Na_2_HPO_4_ buffer (50 mM, pH 5.0–8.5) and Na_2_HPO_4_–KH_2_PO_4_ buffer (50 mM, pH 6.0–7.5). **b** Optimum temperature of AmyZ1. The assays were measured at temperatures from 20 to 70 °C in Na_2_HPO_4_–KH_2_PO_4_ buffer (50 mM, pH7.0). **c** Effect of pH on enzyme stability. The purified enzyme was incubated in 50 mM Na_2_HPO_4_–KH_2_PO_4_ buffer (pH 6.0–7.0) at 30 °C and the remaining activities were measured at appropriate intervals. **d** Effect of temperature on enzyme stability. The purified enzyme was incubated at 20–35 °C in Na_2_HPO_4_–KH_2_PO_4_ buffer (50 mM, pH 6.5). The remaining activities was determined at appropriate intervals. All the results were the average of triplicate experiments

With raw rice starch as the substrate, AmyZ1 exhibited the highest activity at pH 7.0 in both citrate–Na_2_HPO_4_ and Na_2_HPO_4_–KH_2_PO_4_ buffers (Fig. 2a). However, AmyZ1 displayed higher specific activity in Na_2_HPO_4_–KH_2_PO_4_ buffer than that in citrate–Na_2_HPO_4_ buffer. As a result, the Na_2_HPO_4_–KH_2_PO_4_ buffer was selected in the following tests. As shown in Fig. 2a, AmyZ1 possessed more than 87% maximum activity in the range of pH 6.0–7.5 in 50 mM Na_2_HPO_4_–KH_2_PO_4_ buffer.

AmyZ1 showed the highest activity at 35 °C and maintained more than 80% of residue activity at the range from 30 to 50 °C (Fig. 2b). Furthermore, AmyZ1 showed more than 40% of the highest activity at 20 °C, indicating the “cold-active” catalytic ability (Fig. 2b). It has been widely believed that most marine α-amylases possess a narrow range of optimum pHs and optimum temperatures [29]. However, AmyZ1 retained most of its activity in a broad pH and temperature range, implicating its great application potential in the starch processing industry.

**Fig. 3:**
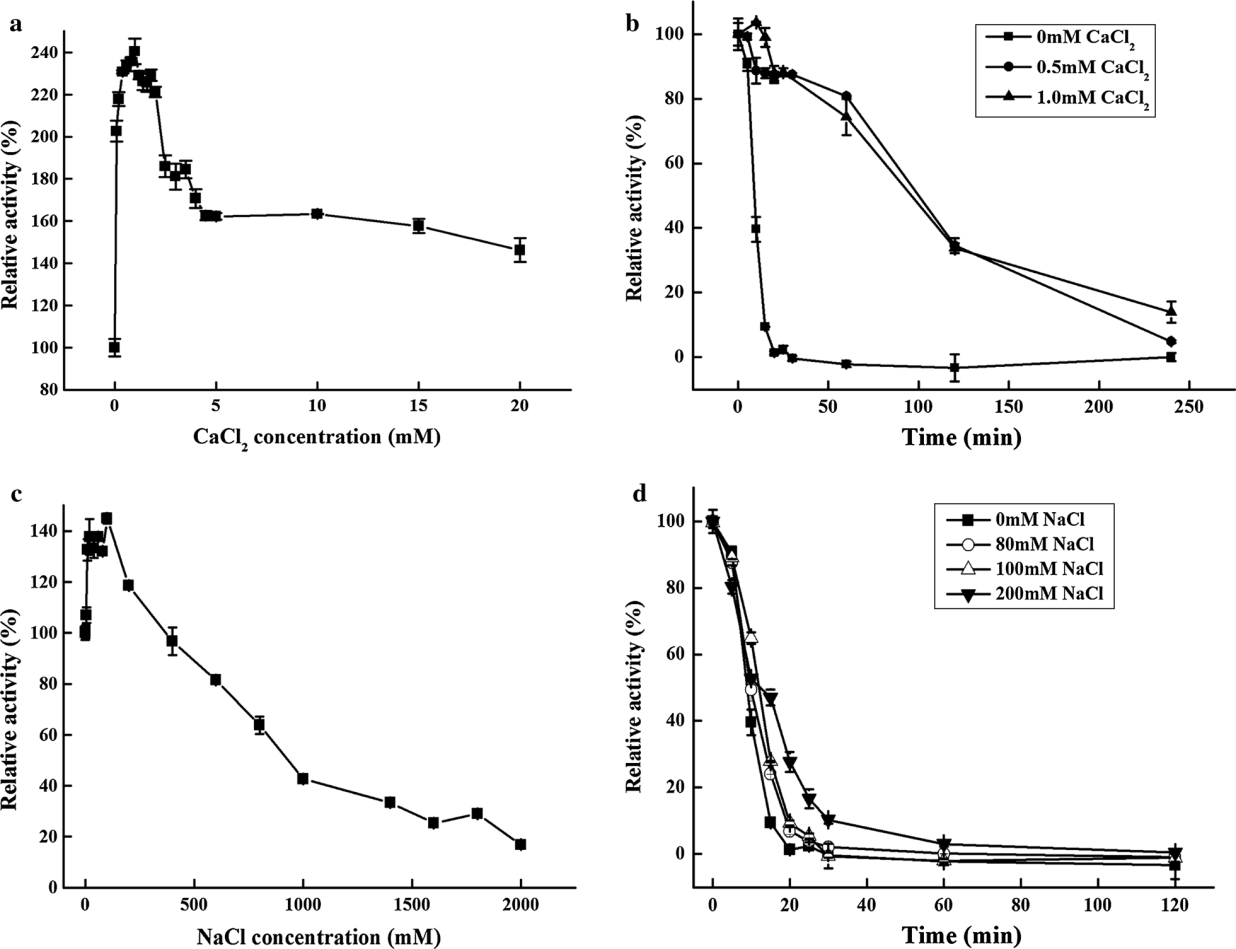
Effects of CaCl_2_ and NaCl on AmyZ1 activity (**a**, **c**) and stability (**b**, **d**). For the effect on enzyme activity, the assays were carried out at 35 °C with additional supplement of CaCl_2_ or NaCl. For the effect on enzyme stability, the assays were performed by incubating the enzyme at 35 °C in Na_2_HPO_4_–KH_2_PO_4_ buffer (50 mM, pH 7.0) containing CaCl_2_ or NaCl. The residual activities were determined at various time intervals. All the results were the average of triplicate experiments

AmyZ1 was more stable at pH 6.5 than that at pH 6.0 and pH 7.0 (Fig. 2c). It exhibited poor pH- and thermo-stability in the absence of Ca^2+^, with about 60% of the activity was lost within 10 min at 35 °C and pH 6.5 (Fig. 2d). The half-life of AmyZ1 at 30 °C and pH 6.5 was only about 12 min (Fig. 2d). In comparison, the introduction of Ca^2+^ significantly improved AmyZ1 catalytic activity and stability. AmyZ1 activity was enhanced to a maximum value of more than 2.4-fold in the presence of 1.0 mM Ca^2+^ compared to that without Ca^2+^ addition at 35 °C (Fig. 3a). Furthermore, the half-life of AmyZ1 at 35 °C was increased to approximately 100 min, approaching 10-fold longer than the time without Ca^2+^ addition (Fig. 3b). When incubated at 30 °C, AmyZ1 retained more than 50% residual activity after 15 h incubation in the presence of 1.0 mM Ca^2+^ (Additional file 5: Fig. S4). Thus, AmyZ1 was relatively stable at lower temperatures, similar to some marine-derived α-amylases [30–33]. These enzymes may be beneficial to be applied in the processes that enzyme is required to be completely inactivated with increasing temperatures [30, 34].

On the other hand, similar to AmyZ1, Ca^2+^ could improve the activity and thermostability of some α-amylases. For example, the α-amylases from *B. licheniformis* [6] and *Bacillus stearothermophilus* [35] displayed improved activity and stability in the presence of Ca^2+^. Based on the crystal structure of α-amylase from *B. licheniformis*, Ca^2+^ was speculated to play an important role in establishing a Ca^2+^–Na^+^–Ca^2+^ connection in the interior of domain B, and stabilizing the architecture of the catalytic cleft [36–38]. Furthermore, the presence of an extra Ca^2+^-binding region at the interface between domains A and C is also believed to be responsible for a higher stability of the enzyme [38].

Other metal ions such as Na^+^ and K^+^ also stimulated the activity of AmyZ1 at concentrations used in our test. For instance, Na^+^ stimulated AmyZ1 activity with a maximal increase of approximately 1.4-fold, whereas the enzyme stability was improved slightly (Fig. 3c, d). While Mg^2+^, Zn^2+^, Mn^2+^, and Cu^2+^ drastically impaired the enzyme activity (Additional file 6: Table S2). Generally, Cu^2+^ and Zn^2+^ were reported to be inhibitors of α-amylases, such as the enzymes from *Exiguobacterium* sp. [39], *B. licheniformis* [40], *B. subtilis* [41], and *Luteimonas abyssi* [42]. The chelating agent EDTA showed an inhibitory effect on the activity of AmyZ1, further supporting the fact that AmyZ1 was a metalloenzyme.

### Substrate specificity

The substrate specificity of AmyZ1 towards raw starches was investigated using various types of substrates (Table 1). Although AmyZ1 showed negligible activities toward pullulan, α-cyclodextrin, and β-cyclodextrin, it could hydrolyze a broad range of raw starch granules including A, B, and C types. Particularly, different from most α-amylases that preferred wheat and corn raw starches as the favorite substrates [2], AmyZ1 preferentially hydrolyzed raw rice starch. It exhibited the highest specific activity of 12,621 ± 196 U/mg towards rice raw starch, followed by corn and wheat raw starch. The *K*_m_ and *V*_max_ of AmyZ1 were 8.85 ± 0.44 mg/mL and 17,837 ± 440 U/mg using raw rice starch as the substrate. The marine α-amylase AmyP also preferred rice raw starch as the substrate [2]. However, the specific activity of AmyZ1 was about 106-fold higher than that of AmyP (Table 2). AmyZ1 also showed higher specific activity than that of the enzymes derived from bacteria, fungi, and yeast. As shown in Table 2, few enzymes exhibited specific activity exceeding 1000 U/mg toward raw starches, e.g. Gt-amy from the extreme thermophile *G. thermoleovorans* [12], RoAmy from *R. oryzae* [13], and TdAmyA from the thermophilic fungus *T. dupontii* [14]. In addition, these enzymes were optimally active at 50 °C or higher. Obviously, AmyZ1 was superior to them not only because of its higher specific activity but also its lower temperature needed for reactions.

**Table 1:** Substrate specificities of AmyZ1 toward raw starches and soluble starches

	Source	Specific activities (U/mg)	Relative activities (%)
Raw starches	Rice (A type)	12,621 ± 196	100
	Corn (A type)	9055 ± 251	72
	Wheat (A type)	5158 ± 133	41
	Barley (A type)	4872 ± 146	39
	Potato (B type)	2623 ± 239	21
	Bean (C type)	1009 ± 42	8
Soluble starches	Soluble starch	14,815 ± 310	117
	Amylose	14,428 ± 111	114
	Amylopectin	23,626 ± 367	187

**Table 2:** Specific activities of AmyZ1 and other known α-amylases toward soluble starches and raw starches

Source (enzyme name)	Specific activity (U/mg)					Refs
	Soluble starch	Rice	Corn	Wheat	Potato	
Bacteria						
*Bacillus licheniformis* 9945a (BliAmy)	3667	NM	NM	NM	NM	[49]
*Luteimonas abyssi* XH031T (LaaA)	8881	NM	NM	NM	NM	[42]
*Bacillus licheniformis* AS08E (Blamy-I)	1063	NM	NM	NM	NM	[50]
*Bacillus* sp. UEB-S	900	NM	NM	NM	NM	[51]
*Streptomyces badius* DB-1	247	156	148	212	NM	[52]
*Geobacillus thermoleovorans* (Gt-amy)	1723	1049	2076	2371	NM	[12]
*Geobacillus* sp. (Gs4j-amyA)	8600	NM	NM	NM	NM	[17]
*Bacillus amyloliquefaciens*	72	31	45	40	68	[10]
*Aeromonas salmonicida* (AmyASS)	45	52	NM	5	NM	[22]
Marine bacterial metagenomes (AmyP) (unknown marine bacterium)	NM	119	NM	7	12	[2]
*Bacillus acidicola*	1166	875	793	979	NM	[11]
*Bacillus* sp. YX-1	NM	NM	NM	NM	607	[53]
*Corallococcus* sp. strain EGB (AmyM) *sp. Strain EGB*	14,000	NM	NM	NM	NM	[54]
*Pontibacillus* sp. ZY (AmyZ1)	14,815	12,621	9056	5158	2623	This study
Fungi						
*Aspergillus penicillioides*	118	NM	NM	NM	NM	[43]
*Aspergillus oryzae* cmc1	32	NM	NM	NM	NM	[44]
*Aspergillus oryzae* cmc1 mutant M-100(6)	2461	NM	NM	NM	NM	[44]
*Aspergillus oryzae* strain S2 (rAmyA)	5150	NM	NM	NM	NM	[45]
*Rhizopus oryzae* (RoAmy)	1123	1044	1449	1494	1089	[13]
*Thermomyces dupontii* (TdAmyA)	2814	2339	2709	2417	1315	[14]
Yeast						
*Cryptococcus flavus* (rAmy1)	4165^a^	NM	NM	NM	NM	[46]
*Cryptococcus flavus*	843^b^	NM	NM	NM	NM	[47]
*Cryptococcus* sp. S-2	2539	NM	NM	NM	NM	[9]
*Saccharomycopsis fibuligera* (Sfamy KZ)	120	91^c^	98^d^	NM	NM	[48]

Enzyme activity was measured by DNS method. One unit of amylase activity was defined as the amount of enzyme needed to release 1 μmol of reducing sugars as maltose per minute

*NM* not mentioned

^a^One unit was defined as the amount of enzyme necessary to produce 1 mg glucose equivalent/min

^b^One unit of amylase activity was defined as the amount of enzyme necessary to hydrolyze 0.1 mg starch per minute

^c,d^The substrates were boiled

AmyZ1 showed the specific activities of up to 23,626 ± 367 and 14,428 ± 111 U/mg, respectively, toward amylopectin than amylose, indicating that the enzyme was able to hydrolyze both α-1,4 and α-1,6 glycosidic linkages with high efficiency (Table 1). However, this phenomenon is abnormal for α-amylases because they generally display higher activities toward amylose than that of amylopectin [2, 11, 42, 51, 52]. As listed in Table 2, the specific activity of AmyZ1 toward soluble starch was up to 14,815 ± 310 U/mg, higher than that of AmyM, an α-amylase from the soil bacterium *Corallococcus* sp. Strain EGB, which was reported in 2015 as the most efficient soluble starch hydrolyzing enzyme [54]. Furthermore, AmyM did not exhibit the raw starch hydrolysis ability [54]. The fact that AmyZ1 could efficiently break both α-1,4 and α-1,6 glycosidic linkages may explain why AmyZ1 possessed higher catalytic activity toward soluble starch than other α-amylases do.

### Hydrolysis toward high concentration raw starches

**Fig. 4:**
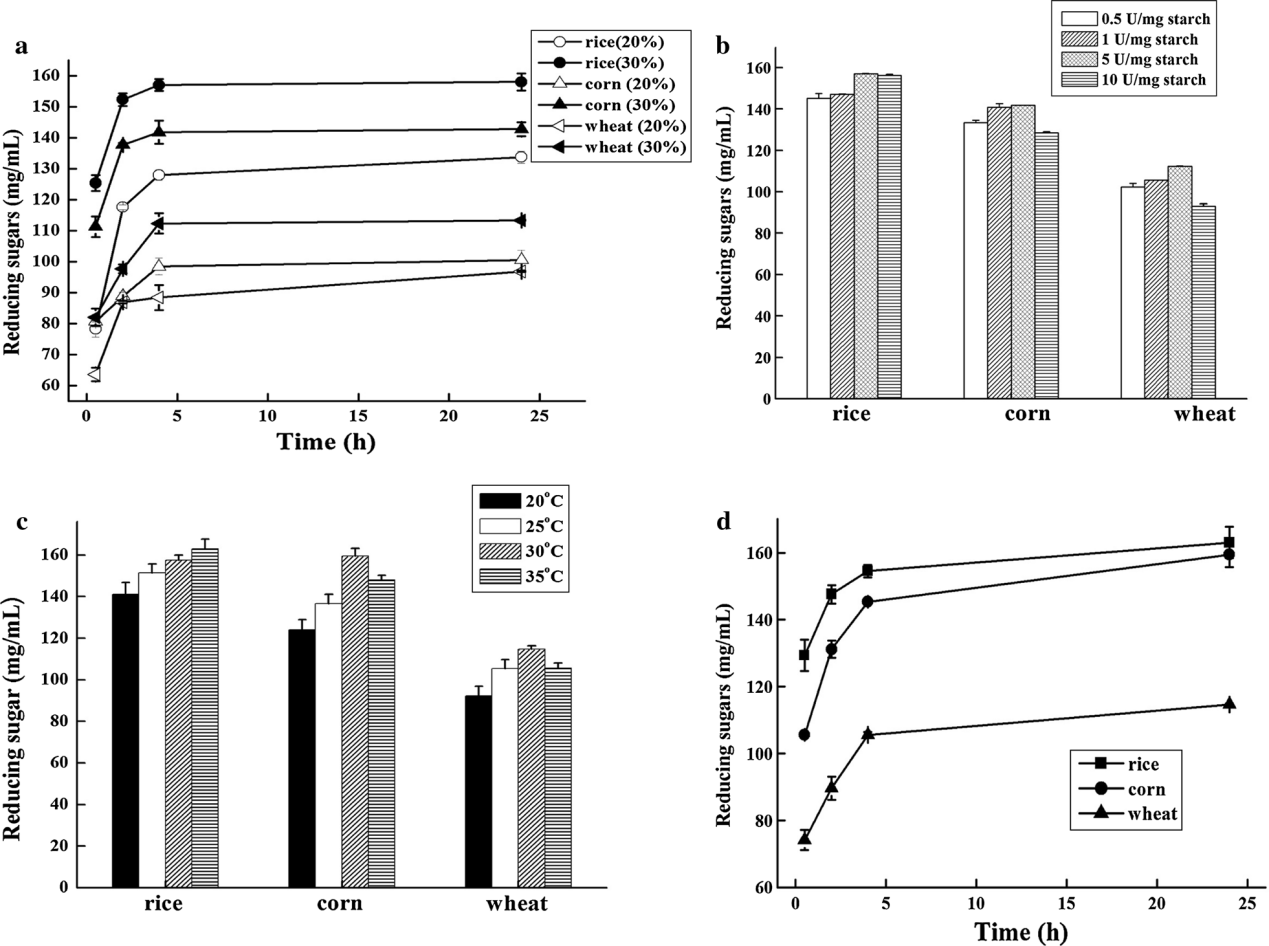
Optimization of hydrolysis conditon toward raw starches from corn, wheat, and rice. **a** Substrate concentrations. The reaction mixture contained 20% or 30% raw starches, and 5 U/mg starch of AmyZ1. The assays was performed at 30 °C and 150 rpm for 24 h. **b** Enzyme dosage. With 30% raw starches as the substrates, the reaction mixture contained 0.5–10 U/mg starch of AmyZ1 as the catalyst. **c** Reaction temperature. The reaction mixture contained 30% raw starch slurry and appropriate units of AmyZ1. The assays was performed at 20–35 °C. **d** Time course of the reaction catalyzed by AmyZ1. All the results were the average of triplicate experiments

The starch-processing industries usually employ 20–30% concentration starch slurries as the starting substrates [16]. Thus, raw starch hydrolysis property of AmyZ1 was assayed under 30% starch concentration (Fig. 4). After hydrolyzation condition optimization, the AmyZ1 dosages employed were 5 U/mg raw rice or wheat starch, and 1 U/mg raw corn starch. The optimized hydrolyzation temperatures were 35 °C for raw rice starch and 30 °C for raw corn or wheat starch. As shown in Fig. 4d, the hydrolysis process displayed a classical two-phase shape, with a rapid initial reaction phase, followed by a slower stage. The most efficient hydrolysis was obtained within the first 4 h. Only a slight increase in reducing sugars was observed after extending the incubation time from 4 to 24 h. After 4 h incubation, the reducing sugars reached 157.1 ± 1.7 mg/mL, 141.8 ± 3.3 mg/mL, and 112.4 ± 0.2 mg/mL with raw rice, corn, and wheat starch as the substrate, respectively. Correspondingly, the hydrolysis rates were 52.4 ± 2.9%, 47.3 ± 1.1% and 37.5 ± 1.1% for raw rice, corn, and wheat starch.

Due to some limiting factors including the surface morphology and crystalline structure of starch granules [56], only a few α-amylases were reported to possess the ability to hydrolyze raw starch slurries at concentrations higher than 15% (Table 3). The temperature is a critical factor influencing the hydrolysis of raw starch slurries at high concentrations. To reach a higher hydrolyzation efficiency, a relatively higher temperature (≥ 60 °C) is usually employed in starch conversion processes, where some commercial thermophilic α-amylases are used, such as those from *B. amyloliquefaciens* [17], *B. licheniformis* [17], and *G. thermoleovorans* [12, 18] (Table 3). Another way to reach a higher hydrolysis rate is to extend incubation time at a lower temperatures (≤ 50 °C) and using the mesophilic enzymes from *Bacillus* sp. YX-1 [53], *Nesterenkonia* sp. strain F [55], and *Rhizomucor* sp. [56] as the catalysts (Table 3). Neither increasing temperature nor extending incubation time could help to reduce energy consumption and lower the cost of manufacturing. Compared to the enzymes listed above, AmyZ1 offers the advantages of efficiently hydrolyzing raw starches at a lower temperature within a shorter reaction time.

**Table 3:** Hydrolysis rates of AmyZ1 and other known α-amylases with high concentration raw starches as the substrates

Source (enzyme name)	Degree of hydrolysis (%)		Enzyme amount (U mg^−1^ starch)	Temp (°C)	pH	Refs
	Wheat	Corn				
Bacteria						
*Bacillus aquimaris* MKSC 6.2 (BaqA)	NM	1.4% (10% and 24 h)	2^a^	37	6.5	[20]
*Bacillus* sp. YX-1	NM	50% (20% and 12 h)	0.3	50	5.0	[53]
*Geobacillus thermoleovorans* (Gt-amy)	26% (30% and 3 h)	24% (30% and 3 h)	0.1	60	5.0	[12]
*Geobacillus thermoleovorans* (Gt-amyII)	NM	31% (30% and 3 h)	70.0	50	7.0	[18]
*Nesterenkonia* sp. strain F	10% (15% and 48 h)	NM	NM	45	NM	[55]
*Geobacillus* sp. 4j (Gs4j-amyA)	45% (30% and 4 h)	36% (30% and 4 h)	0.5	65	5.5	[17]
*Bacillus amyloliquefaciens* (BAA, Sigma)	50% (30% and 4 h)	15% (30% and 4 h)	0.5	65	5.5	[17]
*Bacillus licheniformis* (BLA, Sigma)	50% (30% and 4 h)	35% (30% and 4 h)	0.5	65	5.5	[17]
*Bacillus licheniformis* (BLA, Sigma)	NM	58% (30% and 5 h)	11.5	60	6.5	[16]
*Bacillus licheniformis* ATCC9945a (BliAmy)	NM	58% (30% and 5 h)	11.5	60	6.5	[16]
*Bacillus licheniformis* (BLA, Sigma)	35% (30% and 4 h)	43% (30% and 4 h)	1.0	30	7.0	This study
*Pontibacillus* sp. ZY (AmyZ1)	37% (30% and 4 h)	47% (30% and 4 h)	5.0^b^ or 1.0^c^	30	7.0	This study
Fungi and other						
*Rhizomucor* sp. (RA)	NM	75% (31% and 96 h)^d^	15.5	32	4.5	[56]
Porcine pancreas (PPA)	NM	32% (31% and 96 h)^d^	15.5	37	7.0	[56]

*NM* not mentioned

^a^μg/mg

^b^With raw wheat starch as the substrate

^c^With raw corn starch as the substrate

^d^Total solubilized sugars were measured in the supernatant by the orcinol sulfuric method

On the other hand, less enzyme unit of AmyZ1 was needed to achieve better hydrolyzation towards high concentration raw corn starches. In comparison, more enzyme units were required in the reaction mixture to obtain higher hydrolysis rates, such as 70.0 U/mg starch of Gt-amyII from *G. thermoleovorans*, 15.5 U/mg starch of α-amylase from *Rhizomucor* sp., and 11.5 U/mg starch of enzyme from *B. licheniformis* (Table 3). Furthermore, due to its high specific activity, less protein of AmyZ1 was required in the reaction. In this context, the usage of AmyZ1 would help to reduce the dosage and cost of the enzyme.

**Fig. 5:**
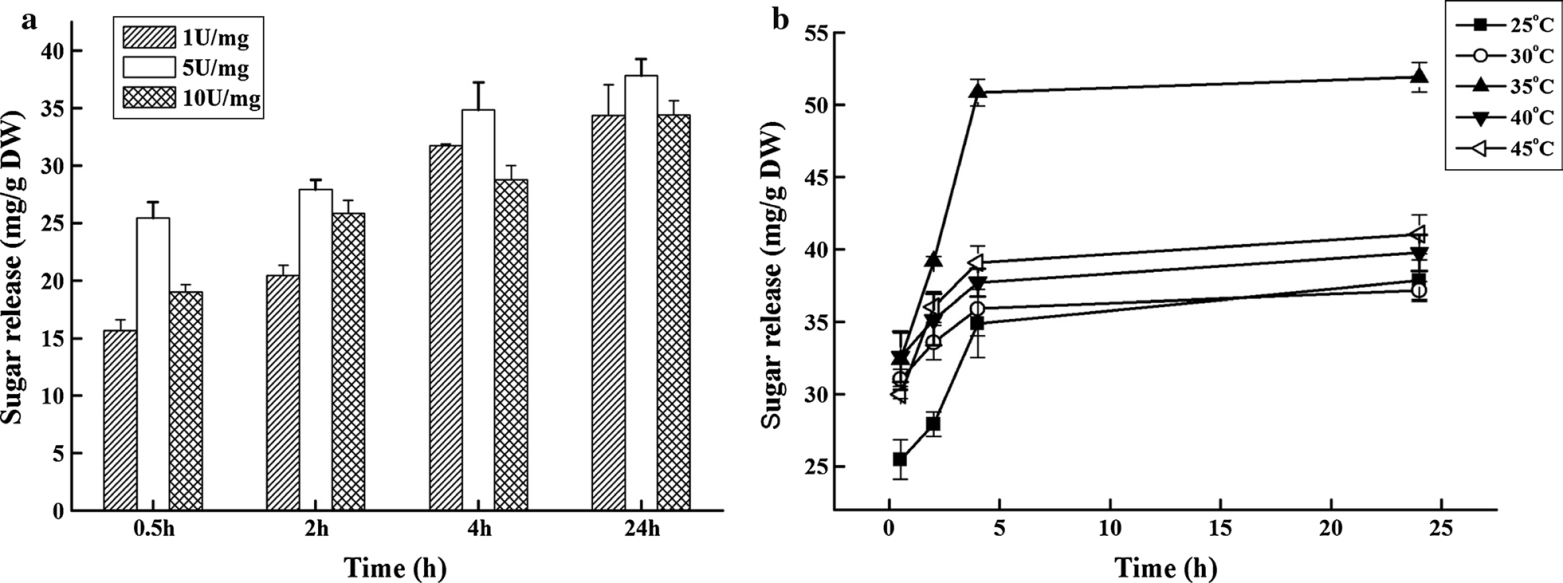
Optimization of hydrolysis condition toward raw starch from microalgae. **a** Enzyme dosage. The reaction mixture contained 1–10 U/mg biomass of AmyZ1 and 5% microalgae biomass. After incubated at 25 °C, the released sugars were measured by the DNS method. **b** Reaction temperature. The reaction mixture was incubated at 25–45 °C, containing 5% microalgae biomass and 5 U/mg biomass of AmyZ1. All the results were the average of triplicate experiments

Although carbohydrate-rich feedstocks are currently used as raw materials for bioethanol production, algae are considered to be the future feedstock because of their high carbohydrate content and absence of lignin compared to higher plants [57, 58]. *Chlorella* sp. has been recognized as one of the best feedstock candidates for bioethanol production, because of its high starch and cellulose content [59]. When AmyZ1 was used to hydrolyze the pretreated *C. pyrenoidosa*, 50.9 ± 0.9 mg/g DW (dry weight of the biomass) of reducing sugars were produced after 4 h incubation at 35 °C with an enzyme dosage of 5 U/mg biomass (Fig. 5b). The reducing sugars released by AmyZ1 approached to the results of acid hydrolysis of *Chlorella sorokiniana* as reported by Hernández et al. [57], although it was lower than that of the combination of acid hydrolysis followed by enzymatic hydrolysis or the combination of various commercial enzymes as a compounded catalyst [57, 59].

### Efficient hydrolysis of raw corn starch with AmyZ1 and the commercial glucoamylase

**Fig. 6:**
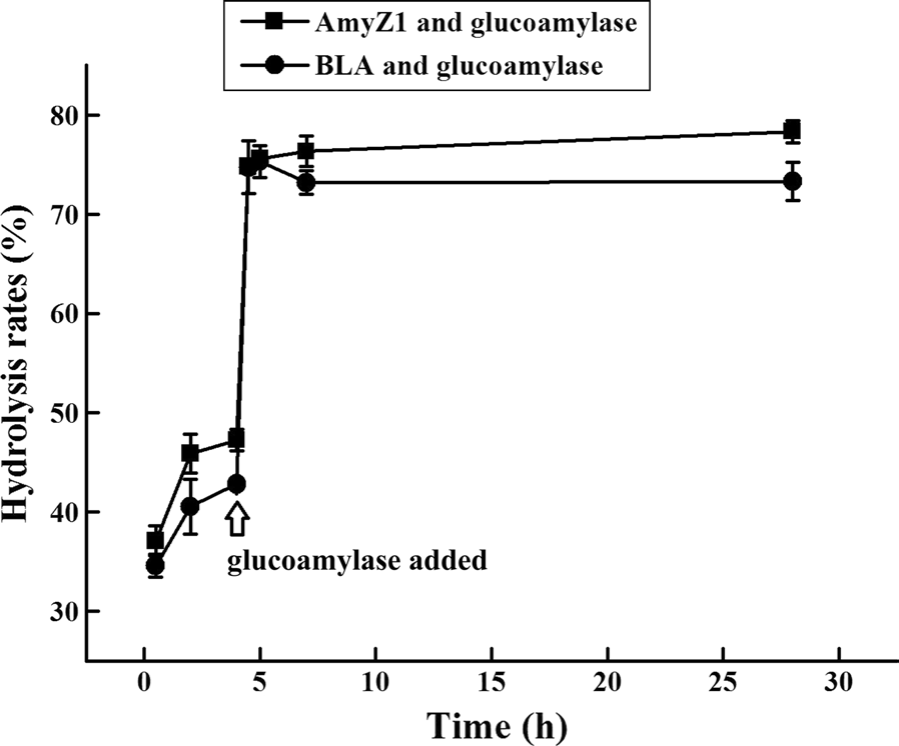
Hydrolysis of raw corn starch by AmyZ1 and the commercial glucoamylase. AmyZ1 (1 U/mg raw starch) was added to 30% raw corn starch slurries. After incubated at 30 °C for 4 h, the mixture was supplemented with the commercial glucoamylase (5 U/mg raw starch), and sequentially incubated at 55 °C for another 24 h. The commercial α-amylase from *Bacillus licheniformis* (BLA) was used as control. All the results were the average of triplicate experiments

AmyZ1 was used to hydrolyze raw corn starch without the gelatinization process of starch. After incubation at 30 °C for 4 h, AmyZ1 hydrolyzed 47.3 ± 1.1% of the starch in raw corn flour (Fig. 6). After the mixture was further treated with commercial glucoamylase from *Aspergillus niger* for 0.5 h, the hydrolysis rate reached 74.8 ± 0.7% and reached 78.3 ± 1.1% after 28 h incubation. In comparison, the commercial BLA showed a hydrolysis rate of 42.8 ± 0.5% on raw corn starch after incubation at 30 °C for 4 h. Furthermore, although the dosages of AmyZ1 and BLA were 1 U/mg raw starch, they are different in protein concentration. Due to a higher specific activity, only 0.13 mg AmyZ1 was added into 30% raw starch slurry, whereas 75 mg of BLA was needed to reach the same activity. α-Amylases from *B. licheniformis* are considered as thermostable enzymes and widely used in starch liquefaction process [6]. Some literatures also reported that the α-Amylases from this genus possessed potency of raw starch digesting [6, 16]. However, when compared with commercial α-amylase BLA, AmyZ1 exhibited higher efficiency towards 30% raw corn starch, with less amount of AmyZ1 required. These properties of AmyZ1 bring more savings to the manufacturers and eventually, the consumers.

Conventionally, raw corn starch processed in bioethanol industries is sequentially gelatinized at about 100 °C, liquefied with thermophilic α-amylase at 95–105 °C for 2–3 h, and treated with glucoamylase at 50–60 °C for about 50 min. These procedures not only require a large amount of energy input, but also give a low yield, with only approximately 50% of corn starch hydrolyzed within about 3–4 h (personal communication with a technical director from Anhui ANTE food Co., Ltd.). When using AmyZ1 paired with the commercial glucoamylase, about 75% of raw starch was hydrolyzed within 4.5 h at 30 °C, without the need of gelatinization step. As a result, the combination of AmyZ1 and glucoamylase will sharply decrease the energy cost and eventually the production cost.

### Action pattern of AmyZ1 on raw starches

To further explore the action pattern of AmyZ1 on raw starches, digested raw starch granules were visualized with a Scanning Electron Microscope (SEM) (Additional file 7: Figure S5). The untreated starch granules remained intact and smooth. However, raw rice starch displayed inhomogeneous holes with different sizes, depth, and width after enzymatic hydrolysis. For raw wheat and corn starches, deeper and smaller holes were observed on the surface of the starch granules. SEM images confirmed that AmyZ1 hydrolyzed the raw starch granules in a random attack mode.

The soluble hydrolysis end products were analyzed using HPLC with 30% raw starches as the substrates (Additional file 8: Table S3). The end products of raw rice starch after hydrolysis were similar to those from raw corn starch, with maltose (G2), maltotriose (G3), and maltopentaose (G5) as the predominant composition, followed by glucose (G1) and maltotetraose (G4). These results suggested that AmyZ1 was a classical saccharifying-type α-amylases, same to those from *B. licheniformis* NH1 [40] and *G. thermoleovorans* [17]. On the other hand, when raw wheat starch was used as the substrate, G2 was the major final oligosaccharide, accounting for approximately 90% of the end products. This was distinct from previous studies of α-amylases on raw wheat starch, of which the end products were various maltooligosaccharides [12, 50, 52]. The action pattern of AmyZ1 towards raw wheat starch makes it as a maltose-forming enzyme, like the enzyme from *B. acidicola* [11]. According to Roy et al., high maltose-forming enzymes are of high demands in the baking industry [50].

## Conclusions

A novel α-amylase AmyZ1 with raw starch hydrolysis ability was cloned from a sediment bacterium *Pontibacillus* sp. ZY. Remarkably, AmyZ1 possessed significantly higher specific activity toward raw starches than other reported α-amylases. In addition to that, AmyZ1 efficiently hydrolyzed raw starches from terrestrial plants and that derived from microalgae. Considering its rapid hydrolysis toward raw starches at a lower temperature, AmyZ1 is undoubtedly a promising candidate for enzymatic hydrolysis toward high concentration raw starch slurries.

## Methods

### Materials and chemicals

The sediment sample from Yongxing island in the South China Sea (sampling site: 16°8′50″N, 112°2′60″E) was collected in Sep 2016 and stored at − 20 °C until use. Soluble starch, amylose, amylopectin, α-cyclodextrin, and β-cyclodextrin were obtained from Sigma Chemical Co. (St. Louis, MO, USA). Rice, corn, and wheat were purchased from the local supermarket (RT-Mart, Hefei, China). They were crushed using food grinder and milled with mortar. The algae powder of *C. pyrenoidosa* was purchased from Guangyu Biological Technology Co., Ltd. (Shanghai, China). The commercial glucoamylase of *A. niger* was purchased from Aladdin Biological Technology Co., Ltd. (Shanghai, China). The commercial α-amylase from *B. licheniformis* (BLA, named Termamyl^®^ 120) was obtained from Sigma Chemical Co. (St. Louis, MO, USA). Other chemicals and reagents were of analytical grade.

### Screening for positive clones with amylolytic activity

One gram of wet sediment was mixed with 9 mL of sterilized seawater, and was shaken at 200 rpm, 15 °C for 2 h. The suspension was subjected to the standard dilution-to-extinction method to 10^−6^. Aliquots of 100 μL dilutions were spread on the screening agar plates (contained 0.2% soluble starch, 0.5% tryptone, 0.1% yeast extract, 3.3% synthetic sea salt, and 1.5% agar) and incubated at 15 °C for 1–3 days. Then the clones were flooded with Lugol’s iodine solution. Positive clones were screened out by the formation of a halo around the clones against the purple background.

### Genomic DNA extraction and analysis of 16S rRNA gene

Positive strains were cultured in 5 mL standard synthetic sea salt medium (Sigma-Aldrich) and incubated at 37 °C, 180 rpm for 12 h. Genomic DNA of the positive strains was extracted according to the manufacturer’s instructions of the kit (Sangon Biotech Co., Ltd; Shanghai, China) and was used as the template. Amplification of the 16S rRNA gene was performed using eubacteria primers of Bact-27F (5′-AGAGTTTGATCMTGGCTCAG-3′) and Bact-1492R (5′-GGTTACCTTGTTACGACTT-3′). The PCR products were cloned into the pGEM-T vector (Promega Corporation, WI, USA) and sequenced (Sangon Biotech Co., Ltd.; Shanghai, China). Then, the Blastn (https://www.ncbi.nlm.nih.gov/) search was carried out to determine the most closely related species.

### Cloning, expression, denaturation and renaturation of AmyZ1

To clone *AmyZ1* from *Pontibacillus* sp. ZY genome, a degenerate primer pair of AmyF (5′-catatgYTNGGNATNWSNTTYGTNYTN-3′, *Nde* I digestion site underlined) and AmyR (5′-ctcgagYTTYTGYTTRTANACNSWNACNSW-3′, *Xho*I digestion site underlined) were designed according to the α-amylase (WP_036770168) from *P. halophilus* JSM 076056. After digested with *Nde*I and *Xho*I, the PCR product was ligated into pET22b(+) (Novagen, Madison, WI) to generate pET22b(+)-*AmyZ1*.

*Escherichia coli* BL21 (DE3) containing pET22b (+)-*AmyZ1* was cultivated in 1 L Luria Broth containing 100 mg/L ampicillin at 37 °C until *OD*_600_ reached 0.6. Protein expression was induced by the addition of 0.2 mM IPTG and the culture was further incubated at 150 rpm and 37 °C for 4 h. Cells were collected at 4 °C by centrifugation at 8000×*g* for 10 min and resuspended in cold Tris–HCl buffer (50 mM, pH 8.0). Then the cells were disrupted by sonication, followed by centrifugation at 10,000×*g* and 4 °C for 30 min to isolate AmyZ1 inclusion bodies.

To refold the protein, AmyZ1 inclusion bodies from 1 L cell cultures were dissolved in 75 mL Tris–HCl buffer (50 mM, pH 8.0) containing 8 M urea. Then dH_2_O supplemented with 10 mM CaCl_2_ was added until the final concentration of urea reached 1 M. After kept at 4 °C for 10 h, the supernatant was pooled and dialyzed against Na_2_HPO_4_–KH_2_PO_4_ buffer (50 mM, pH 6.5) containing 1 mM CaCl_2_ for overnight. All experiments were performed at 4 °C.

The refolded protein was evaluated by SDS-PAGE with 15% polyacrylamide gel. Protein concentration was determined by BCA method according to the protocol (Thermo Fisher Scientific, Waltham, MA, USA). To determine the molecular mass of native protein, the purified protein was analyzed by Native-PAGE using the precast polyacrylamide gel (4–20%; Bio-Rad Laboratories, Inc.), and the unstained protein marker as the standard (Thermo Fisher Scientific, Waltham, MA, USA).

### Bioinformatic analysis of AmyZ1

The presence of a putative signal peptide was predicted using SignalP 4.0 program (http://www.cbs.dtu.dk/services/SignalP/) and simple modular architecture research tool (SMART, http://smart.embl-heidelberg.de/). To classify AmyZ1 into a subfamily, multiple sequence alignment of AmyZ1 with other GH13 α-amylase sequences was performed using ClustalX 2.0. The phylogenetic tree was constructed by MEGA 7 using the Maximum Likelihood method. The conserved regions of enzymes were displayed using GENEDOC (http://www.psc.edu/biomed/genedoc).

The three-dimensional structure of AmyZ1 was generated using the automated Swiss-Model protein modeling server (http://swissmodel.expasy.org) with α-amylase from *B. licheniformis* (PDB code: 1BLI) as the template. The structures were visualized using Pymol (http://www.pymol.org/).

### Enzyme assay

The enzyme activity of AmyZ1 was determined by measuring the reducing sugars released from the hydrolysis of raw starches base on the dinitrosalicylic acid (DNS) assay. The reaction mixture contained 30 μL enzyme solution, and 570 μL Na_2_HPO_4_–KH_2_PO_4_ buffer (50 mM, pH 7.0) supplemented with 1% raw rice starch and 1 mM CaCl_2_. After incubating the mixture at 35 °C for 10 min, the reaction was stopped by adding 300 μL of DNS. The reaction mixture was then heated in boiling water for 15 min. The amount of reducing sugars released was monitored at 540 nm. One unit of amylase activity was defined as the amount of enzyme needed to release 1 μmol of reducing sugars as maltose per minute under standard assay conditions described above. Assays with heat treated AmyZ1 was used as control.

### Effects of temperature and pH on the activity and stability of AmyZ1

The effect of temperature on AmyZ1 activity was determined at temperatures ranging from 20 to 70 °C using raw rice starch as the substrate. The optimum pH of AmyZ1 was examined in the pH range of 4.0 to 8.5 at 35 °C in citrate–Na_2_HPO_4_ buffer (50 mM, pH 5.0–8.5) and Na_2_HPO_4_–KH_2_PO_4_ buffer (50 mM, pH 6.0–7.5). In the absence of Ca^2+^, the thermostability was determined by incubating the enzyme in Na_2_HPO_4_–KH_2_PO_4_ buffer (50 mM, pH 7.0) at 20–35 °C. At appropriate intervals, the residual activity was measured using the DNS method as mentioned above. The pH stability of AmyZ1 without Ca^2+^ was determined by dispersing the enzyme in Na_2_HPO_4_–KH_2_PO_4_ buffer of pH 6.0, 6.5, and 7.0, and the residual activities were measured at appropriate intervals.

### Effects of metal ions and chemicals on enzyme activity and stability

To evaluate the influence of Ca^2+^ and Na^+^ on the activity of AmyZ1, additional supplement of CaCl_2_ and NaCl was included in the reaction mixture that consisted of raw rice starch, appropriate volume of enzyme, and Na_2_HPO_4_–KH_2_PO_4_ buffer (50 mM, pH 7.0). To determine the thermostability in the presence of Ca^2+^ or Na^+^, the enzyme was mixed with different concentrations of CaCl_2_ or NaCl and incubated at 30 or 35 °C. The residual activities were measured at appropriate intervals.

The effects of other metal ions and chemicals on the activity of AmyZ1 were evaluated under the standard assay conditions with additional supplement of 1, 5 or 10 mM Mn^2+^, Cu^2+^, K^+^, Mg^2+^, Zn^2+^, and EDTA. In control, the reaction mixture contained appropriate volume of enzyme and Na_2_HPO_4_–KH_2_PO_4_ buffer (50 mM, pH 7.0) supplemented with rice raw starch as the substrate. The enzyme activity determined in the control were defined as 100%. In the reaction mixture containing additional metal ions, the enzyme activity was determined and the relative activities were calculated. The relative activities were defined as the activity in the presence of additional metal ions relative to that of control.

### Substrate specificity

The substrate specificity was determined under the standard assay condition using 1% (w/v) raw starch from various origins including rice, corn, wheat, barley, potato, and bean. Soluble starch, amylose, amylopectin, pullulan, α-cyclodextrin, and β-cyclodextrin were also used as the substrates.

The kinetic constants of AmyZ1, including *K*_m_ and *V*_max_, were measured using raw rice starch as the substrate based on the DNS assay. The reaction was performed by incubating the enzyme in Na_2_HPO_4_–KH_2_PO_4_ (50 mM, pH 7.0) supplemented with varying concentrations of raw rice starch (1.0–20 mg/mL). The reaction was performed at 35 °C for 10 min. The kinetic parameters were calculated by fitting the experimental data to Lineweaver–Burt equation of the Michaelis–Menten model using Origin 8.0.

### Hydrolysis condition optimization towards high concentration raw starches

Hydrolysis activity of AmyZ1 toward high concentration raw starch was evaluated using raw starches from terrestrial plants including rice, corn, and wheat, as well as aquatic algae *C. pyrenoidosa*. With raw rice, corn, and wheat starch as the substrates, factors including enzyme unit (0.5–10 U/mg starch), substrate concentration (20% or 30%, w/v), reaction temperature (20–35 °C), and reaction time (1–24 h) were optimized using the single factor analysis. Reactions were carried out in a mixture containing appropriate volume of enzyme, raw starches, and Na_2_HPO_4_–KH_2_PO_4_ buffer (50 mM, pH 7.0) supplemented with 1 mM CaCl_2_.

Specially, when using raw starch from *C. pyrenoidosa* as the substrate, the *C. pyrenoidosa* powder was suspended in Na_2_HPO_4_–KH_2_PO_4_ buffer (50 mM, pH 7.0) to a final concentration of 5% (w/v). The mixture was disrupted by sonication for 30 min at a frequency of 40 kHz and an acoustic power up to 450 W [60]. Five percent (w/v) biomass was incubated with AmyZ1 with the dosage of 1, 5 and 10 U/mg biomass. The mixture was incubated at 25 to 45 °C and the reducing sugars in supernatant were assayed by the DNS method at appropriate intervals.

The extent of raw starch hydrolysis was calculated using the following formula:

$$\begin{aligned} {\text{Hydrolysis rates }}\left( \% \right)\, = & \,\left[ {{{{\text{reducing sugars }}\left( {{\text{mg}} {\text{ mL}}^{ - 1} } \right)} \mathord{\left/ {\vphantom {{{\text{reducing sugars }}\left( {{\text{mg}} {\text{ mL}}^{ - 1} } \right)} {{\text{initial weight of raw starch }}\left( {{\text{mg}} {\text{mL}}^{ - 1} } \right)}}} \right. \kern-0pt} {{\text{initial weight of raw starch }}\left( {{\text{mg}} {\text{mL}}^{ - 1} } \right)}}} \right] \\ & \times \,0. 9\, \times \, 100. \\ \end{aligned}$$

The factor 0.9 (referred to 162/180) is the conversion factor caused by hydrolysis reaction [17].

### Hydrolysis of raw corn starch by AmyZ1 and the commercial glucoamylase

Thirty percent (w/v) raw corn starch slurries were employed to evaluate the combined hydrolysis effect of α-amylase AmyZ1 and the commercial glucoamylase from *A. niger*. The purified AmyZ1 (1 U/mg raw starch) was added to 30% raw corn starch slurries. The reaction mixture was incubated at 30 °C for 4 h with shaking at 150 rpm. Then, the mixture was supplemented with the commercial glucoamylase (5 U/mg raw starch) and sequentially incubated at 55 °C for another 24 h. Furthermore, the commercial α-amylase from *B. licheniformis* (BLA) was used as control to hydrolyze raw corn starch performed as mentioned above. At appropriate intervals, the samples were withdrawn and the reducing sugars were measured by the DNS method using glucose as the standard. The rates of hydrolysis were calculated according to the formula described above.

### Scanning electron microscopy

The raw starch shape before and after hydrolysis was visualized using a Scanning Electron Microscope (SEM, HITACHI S4800, Japan). Briefly, the reaction was conducted using AmyZ1 (5 U/mg starch) and 5% raw starches from various sources including rice, corn, and wheat. After incubation at 30 °C for 30 min, the mixture was centrifuged at 8000×*g* to recover the pellets. Then the pellets were washed with pure ethanol for three times, followed by drying at 35 °C to a constant weight. The samples were fixed on a specimen holder using a silver plate and coated with Pt using Ion Sputter E-1010 at 5.0 kV and 20 mA for 40 s. The specimens were then viewed with SEM.

### Analysis of the hydrolyzed products

The hydrolytic products of AmyZ1 from raw starches were determined using high-performance liquid chromatography (HPLC; Agilent Corp., Palo Alto, CA) equipped with a Carbohydrate ES column (5 μm, 250 × 4.6 mm, Alltech) and an evaporative light scattering detector. The mobile phase was acetonitrile and water (55: 45, v/v) with a flow rate of 1.0 mL/min at 25 °C. Glucose (G1), maltose (G2), maltotriose (G3), maltotetraose (G4), and maltopentaose (G5) were used as standards.

### Nucleotide sequences accession number

The partial 16S rRNA gene sequence of *Pontibacillus* sp. ZY and AmyZ1 amino acid sequence have been deposited in the GenBank database with the accession number MH279661 and AXV43605, respectively.

## Additional files

**Additional file 1: Figure S1.** Alignment of the amino acid sequences of AmyZ1 and other known α-amylases. The four conserved regions (region I-region IV) were boxed. The key catalytic residues were indicated below the sequences by a black triangle. 1BLI: α-amylase from *Bacillus licheniformis*; ADE44086: α-amylase from *Bacillus amyloliquefaciens*; WP_058836133: α-amylase from *Luteimonas abyssi.*

**Additional file 2: Figure S2.** Phylogenic analysis of AmyZ1 and other reported α-amylases of GH13 family. The protein sequences of different subfamilies were retrieved from CAZy database. The tree was built using Maximum Likelihood method of the program MEGA 7. The bootstrap values were calculated based on 1000 replicates.

**Additional file 3: Figure S3.** Homology model structure of AmyZ1. The structure of AmyZ1 was constructed based on its closest structural relative *Bacillus licheniformis* α-amylase (PDB code: 1BLI). The domains were colored as follows: A, red; B, green; C, blue.

**Additional file 4: Table S1.** Denaturation and renaturation of α-amylase AmyZ1.

**Additional file 5: Figure S4.** Effects of NaCl and CaCl_2_ on AmyZ1 stability. The assays were performed by incubating the enzyme at 30 °C in Na_2_HPO_4_–KH_2_PO_4_ buffer (50 mM, pH 7.0) containing additional CaCl_2_ or NaCl. The residual activities were determined at various time intervals.

**Additional file 6: Table S2.** Effects of metal ions on enzyme activity.

**Additional file 7: Figure S5.** Scanning electron microscopy of raw starch granules hydrolyzed by AmyZ1. a, c and e: raw rice, corn, and wheat starch before hydrolysis; b, d and f: raw rice, corn, and wheat starch treated with AmyZ1.

**Additional file 8: Table S3.** Hydrolysis products of raw starches catalyzed by AmyZ1.

## Abbreviations

DW: dry weight of the biomass
BLAST: basic local alignment search tool
SMART: simple modular architecture research tool
DNS: dinitrosalicylic acid
IPTG: isopropyl β-d-1-thiogalactopyranoside
SDS–PAGE: sodium dodecyl sulfate polyacrylamide gel electrophoresis
BLA: the commercial α-amylase of *Bacillus licheniformis*
SEM: scanning electron microscope
HPLC: high-performance liquid chromatography
G1: glucose
G2: maltose
G3: maltotriose
G4: maltotetraose
G5: maltopentaose
