# Supplementary figures and images for: AmyZ1: a novel α-amylase from marine bacterium Pontibacillus sp. ZY with high activity toward raw starches
Source: Biotechnol Biofuels. 2019 Apr 23;12:95. doi: 10.1186/s13068-019-1432-9 (PMC6477751; doi:10.1186/s13068-019-1432-9)

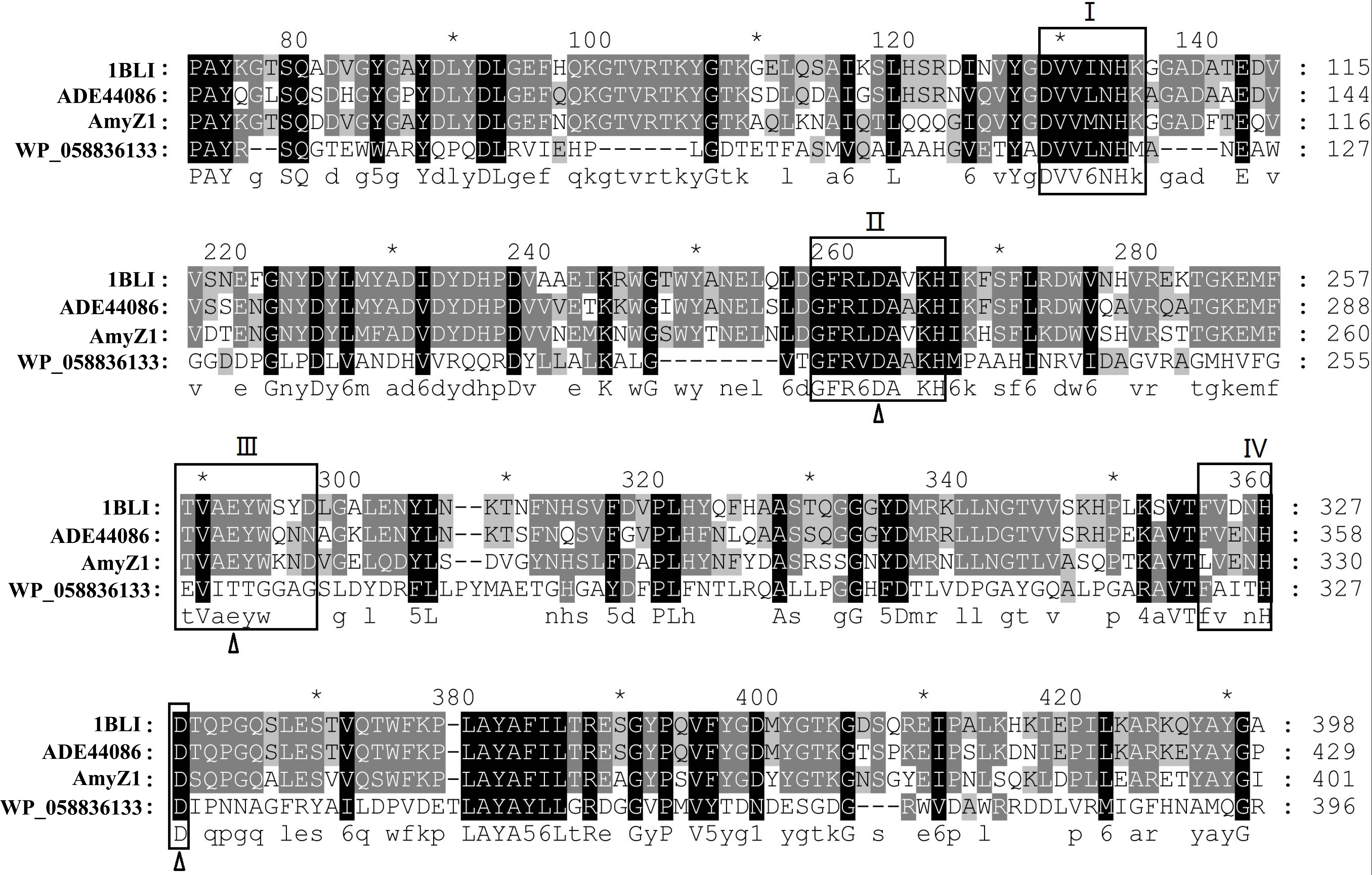

Supplement: Supplementary file 1 — Additional file 1: Figure S1. Alignment of the amino acid sequences of AmyZ1 and other known α-amylases. The four conserved regions (region I-region IV) were boxed. The key catalytic residues were indicated below the sequences by a black triangle. 1BLI: α-amylase from Bacillus licheniformis; ADE44086: α-amylase from Bacillus amyloliquefaciens; WP_058836133: α-amylase from Luteimonas abyssi. [file 13068_2019_1432_MOESM1_ESM.jpg]

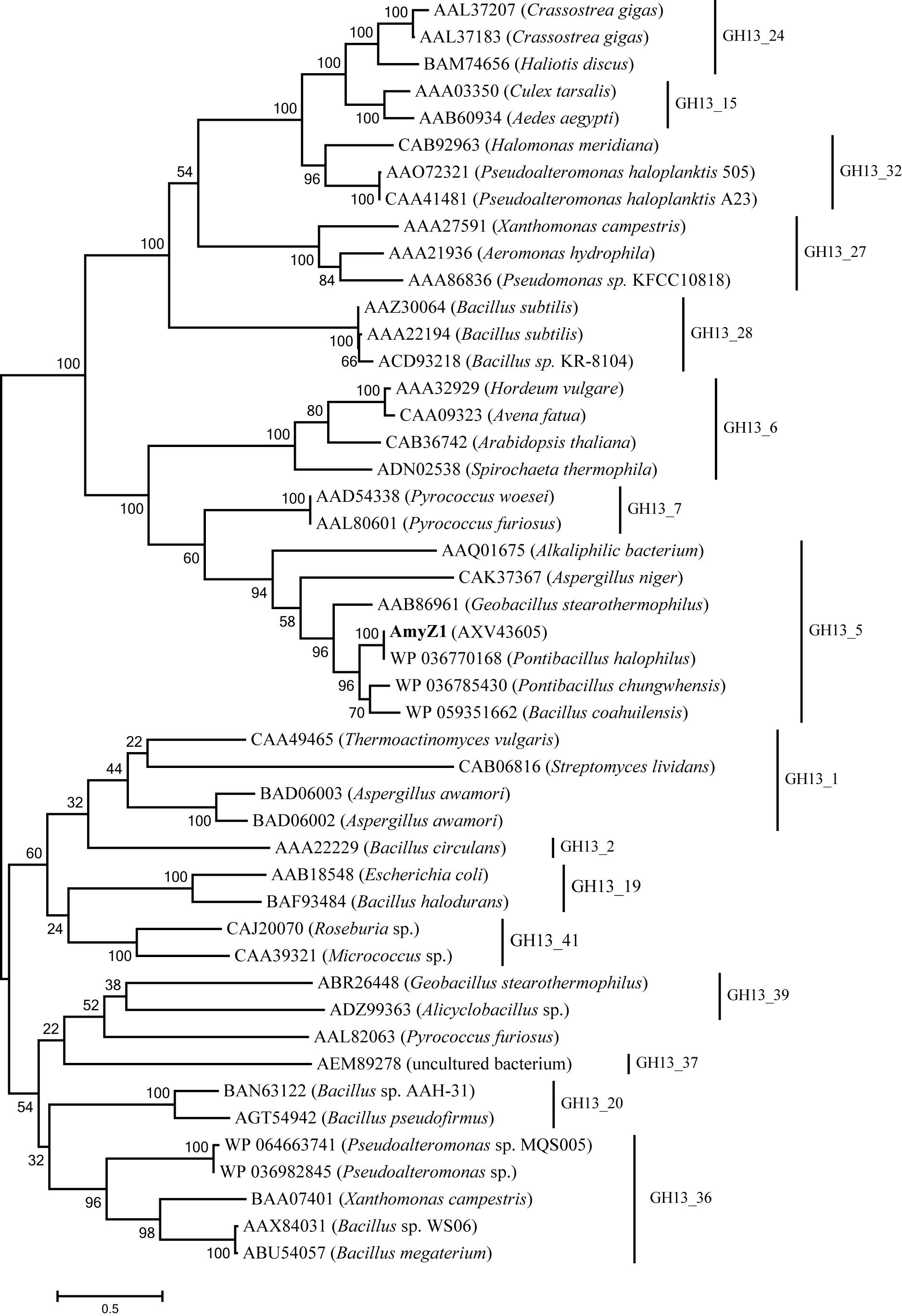

Supplement: Supplementary file 2 — Additional file 2: Figure S2. Phylogenic analysis of AmyZ1 and other reported α-amylases of GH13 family. The protein sequences of different subfamilies were retrieved from CAZy database. The tree was built using Maximum Likelihood method of the program MEGA 7. The bootstrap values were calculated based on 1000 replicates. [file 13068_2019_1432_MOESM2_ESM.jpg]

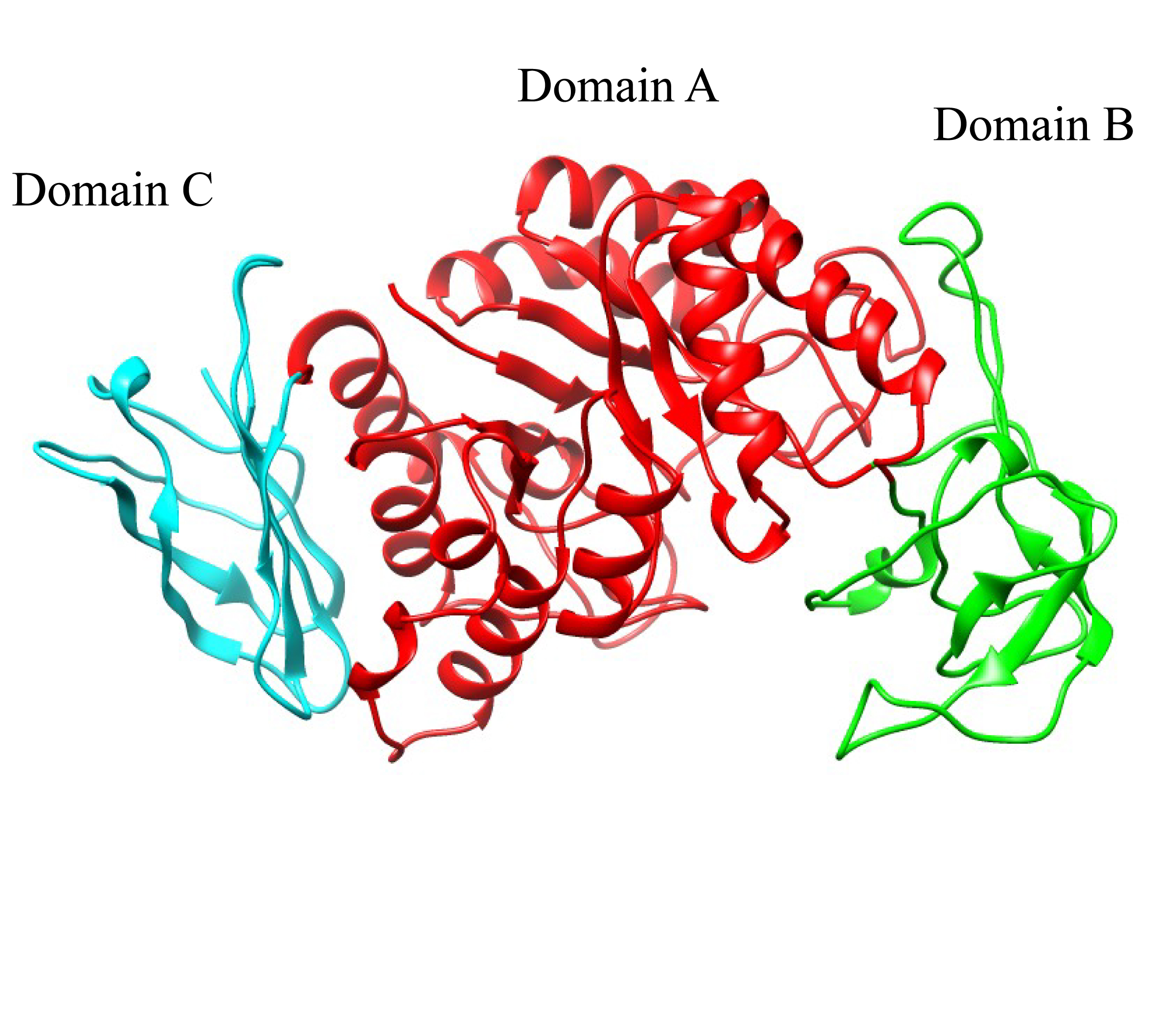

Supplement: Supplementary file 3 — Additional file 3: Figure S3. Homology model structure of AmyZ1. The structure of AmyZ1 was constructed based on its closest structural relative Bacillus licheniformis α-amylase (PDB code: 1BLI). The domains were colored as follows: A, red; B, green; C, blue. [file 13068_2019_1432_MOESM3_ESM.jpg]

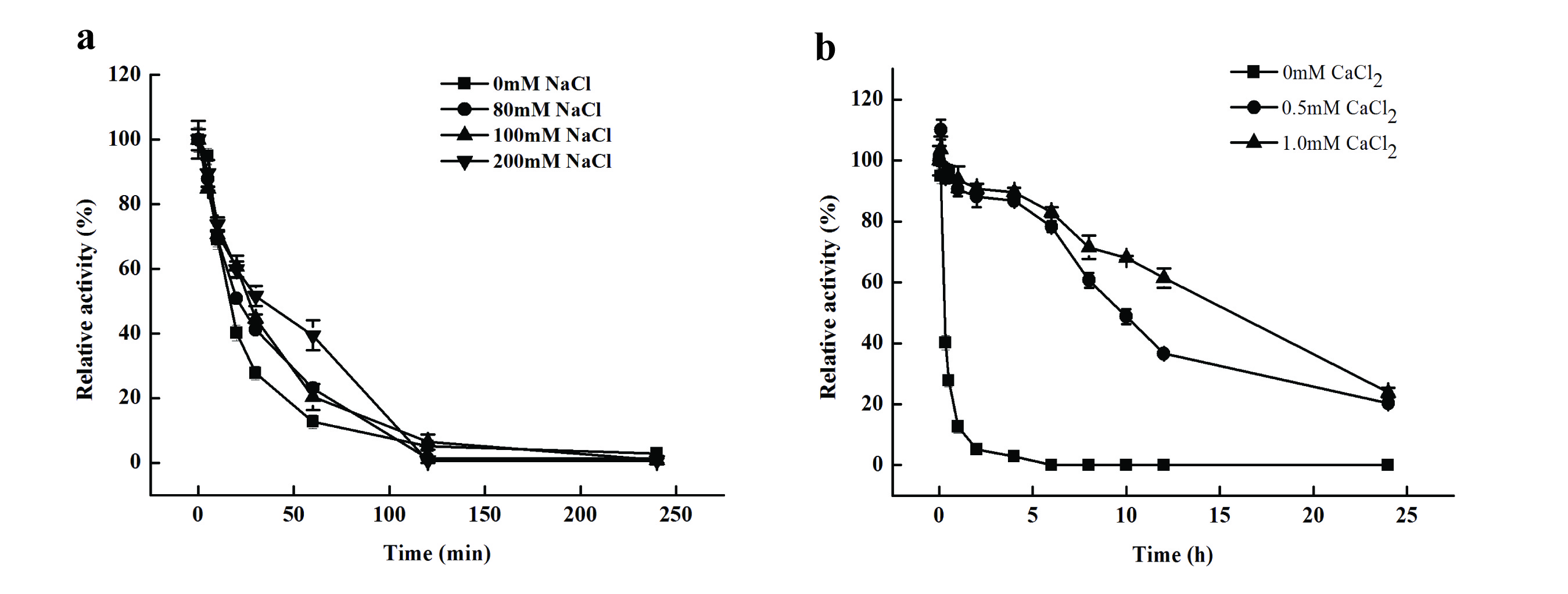

Supplement: Supplementary file 5 — Additional file 5: Figure S4. Effects of NaCl and CaCl2 on AmyZ1 stability. The assays were performed by incubating the enzyme at 30 °C in Na2HPO4–KH2PO4 buffer (50 mM, pH 7.0) containing additional CaCl2 or NaCl. The residual activities were determined at various time intervals. [file 13068_2019_1432_MOESM5_ESM.jpg]

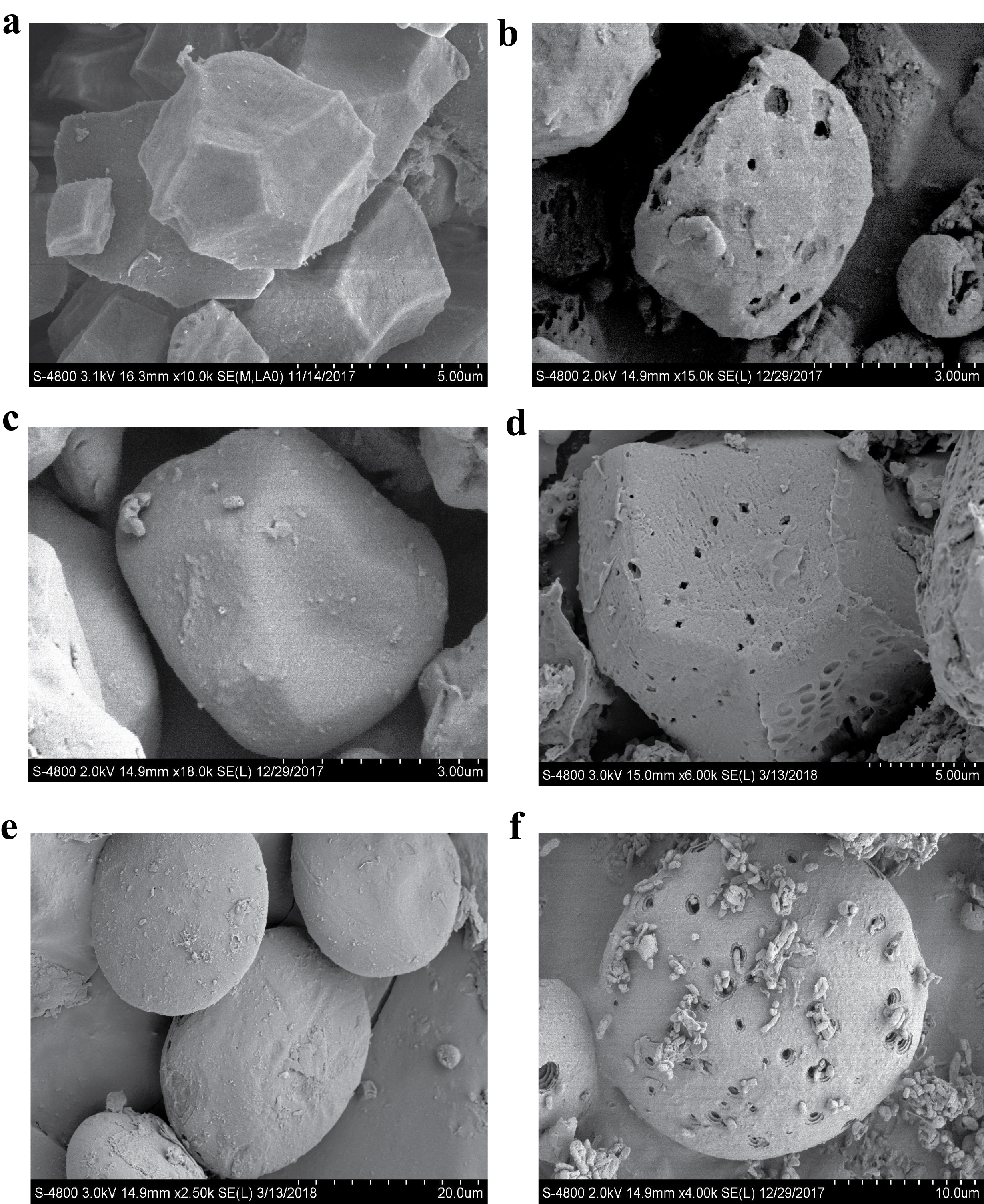

Supplement: Supplementary file 7 — Additional file 7: Figure S5. Scanning electron microscopy of raw starch granules hydrolyzed by AmyZ1. a, c and e: raw rice, corn, and wheat starch before hydrolysis; b, d and f: raw rice, corn, and wheat starch treated with AmyZ1. [file 13068_2019_1432_MOESM7_ESM.jpg]
