# Supplementary material for: AmyZ1: a novel α-amylase from marine bacterium Pontibacillus sp. ZY with high activity toward raw starches
Source: Biotechnol Biofuels. 2019 Apr 23;12:95. doi: 10.1186/s13068-019-1432-9 (PMC6477751; doi:10.1186/s13068-019-1432-9)
Supplement: Supplementary file 4 — Additional file 4: Table S1. Denaturation and renaturation of α-amylase AmyZ1. [file 13068_2019_1432_MOESM4_ESM.docx]

| Steps | Total volume (mL) | Total activity (U) | Total protein (mg) | Specific activity  (U/mg) | Yield (%) | Fold |
| --- | --- | --- | --- | --- | --- | --- |
| Denaturation | 30 | 0 | 105.1 | 0 | 100 |  |
| Renaturation | 240 | 289528.9 | 42.8 | 9481.7 | 40.7 | 1 |
| Dialysis | 240 | 278906.7 | 30.9 | 12621.3 | 29.4 | 1.3 |

Table S1 Denaturation and renaturation of α-amylase AmyZ1
