# Supplementary material for: AmyZ1: a novel α-amylase from marine bacterium Pontibacillus sp. ZY with high activity toward raw starches
Source: Biotechnol Biofuels. 2019 Apr 23;12:95. doi: 10.1186/s13068-019-1432-9 (PMC6477751; doi:10.1186/s13068-019-1432-9)
Supplement: Supplementary file 6 — Additional file 6: Table S2. Effects of metal ions on enzyme activity. [file 13068_2019_1432_MOESM6_ESM.docx]

| Metal ions | Final concentration | Relative enzyme activity (%) |
| --- | --- | --- |
| K^+^ | 1mM | 134.90±0.05 |
|  | 5mM | 126.88±0.02 |
|  | 10mM | 123.93±0.06 |
| Mg^2+^ | 1mM | 99.37±0.04 |
|  | 5mM | 85.68±0.01 |
|  | 10mM | 78.11±0.06 |
| Zn^2+^ | 1mM | 60.71±0.02 |
|  | 5mM | 23.35±0.06 |
|  | 10mM | 0 |
| Mn^2+^ | 1mM | 19.12±0.01 |
|  | 5mM | 0 |
| Cu^2+^ | 1mM | 0 |

Table S2 Effects of metal ions on enzyme activity
