# Supplementary material for: AmyZ1: a novel α-amylase from marine bacterium Pontibacillus sp. ZY with high activity toward raw starches
Source: Biotechnol Biofuels. 2019 Apr 23;12:95. doi: 10.1186/s13068-019-1432-9 (PMC6477751; doi:10.1186/s13068-019-1432-9)
Supplement: Supplementary file 8 — Additional file 8: Table S3. Hydrolysis products of raw starches catalyzed by AmyZ1. [file 13068_2019_1432_MOESM8_ESM.docx]

Table S3 Hydrolysis products of raw starches catalyzed by AmyZ1

| Raw starch | Time | G1 (%) | G2 (%) | G3 (%) | G4 (%) | G5 (%) |
| --- | --- | --- | --- | --- | --- | --- |
| Rice | 0.5h | 9.2 | 26.1 | 30.7 | 7.8 | 26.2 |
|  | 2h | 8.2 | 20.8 | 28.9 | 7.2 | 34.9 |
|  | 4h | 7.9 | 26.3 | 31.4 | 5.9 | 28.5 |
| Corn | 0.5h | 11.4 | 29.3 | 35.0 | 4.8 | 19.5 |
|  | 2h | 12.9 | 26.1 | 33.6 | 6.1 | 21.3 |
|  | 4h | 15.4 | 27.2 | 32.7 | 6.9 | 17.8 |
| Wheat | 0.5h | 8.4 | 91.6 | 0 | 0 | 0 |
|  | 2h | 10.3 | 89.7 | 0 | 0 | 0 |
|  | 4h | 10.8 | 89.2 | 0 | 0 | 0 |
